# Association of Electronic Cigarette Use With Incident Respiratory Conditions Among US Adults From 2013 to 2018

**DOI:** 10.1001/jamanetworkopen.2020.20816

**Published:** 2020-11-12

**Authors:** Wubin Xie, Hasmeena Kathuria, Panagis Galiatsatos, Michael J. Blaha, Naomi M. Hamburg, Rose Marie Robertson, Aruni Bhatnagar, Emelia J. Benjamin, Andrew C. Stokes

**Affiliations:** 1 Department of Global Health, Boston University School of Public Health, Boston, Massachusetts; 2 The Pulmonary Center, Boston University School of Medicine, Boston, Massachusetts; 3 Pulmonary and Critical Care Medicine, Johns Hopkins School of Medicine, Baltimore, Maryland; 4 Ciccarone Center for the Prevention of Cardiovascular Disease, Johns Hopkins Medical Institutions, Baltimore, Maryland; 5 Department of Epidemiology, Johns Hopkins Bloomberg School of Public Health, Baltimore, Maryland; 6 Department of Medicine, Boston University School of Medicine, Boston, Massachusetts; 7 Department of Medicine, Vanderbilt University School of Medicine, Nashville, Tennessee; 8 American Heart Association Tobacco Regulation and Addiction Center, Dallas, Texas; 9 Department of Medicine, University of Louisville, Louisville, Kentucky; 10 Department of Epidemiology, Boston University School of Public Health, Boston, Massachusetts

## Abstract

**Importance:**

Generating robust and timely evidence about the respiratory health risks of electronic cigarettes (e-cigarettes) is critical for informing state and federal regulatory standards for product safety.

**Objective:**

To examine the association of e-cigarette use with incident respiratory conditions, including chronic obstructive pulmonary disease (COPD), emphysema, chronic bronchitis, and asthma.

**Design, Setting, and Participants:**

This prospective cohort study used data from the nationally representative cohort of US adults from the Population Assessment of Tobacco and Health (PATH) study, including wave 1 from 2013 to 2014, wave 2 from 2014 to 2015, wave 3 from 2015 to 2016, and wave 4 from 2016 to 2018. Individuals aged 18 years and older at baseline with no prevalent respiratory conditions were included in the analyses. Analyses were conducted from February to July 2020.

**Exposures:**

e-Cigarette use was assessed by self-reported current use status (never, former, or current) at baseline.

**Main Outcomes and Measures:**

Incident respiratory conditions, including COPD, emphysema, chronic bronchitis, and asthma, as well as a composite respiratory disease encompassing all 4 conditions.

**Results:**

Among 21 618 respondents included in the analyses, 11 017 (491%) were men and 12 969 (65.2%) were non-Hispanic White. A total of 14 213 respondents were never e-cigarette users, 5076 respondents (11.6%) were former e-cigarette users, and 2329 respondents (5.2%) were current e-cigarette users. Adjusted for cigarette and other combustible tobacco product use, demographic characteristics, and chronic health conditions, there was an increased risk of respiratory disease among former e-cigarette uses (incidence rate ratio [IRR], 1.28; 95% CI, 1.09-1.50) and current e-cigarette users (IRR, 1.31; 95% CI, 1.08-1.59). Among respondents with good self-rated health, the IRR for former e-cigarette users was 1.21 (95%CI, 1.00-1.46) and the IRR for current e-cigarette users was 1.43 (95% CI, 1.14-1.79). For specific respiratory diseases among current e-cigarette users, the IRR was 1.33 (95% CI, 1.06-1.67) for chronic bronchitis, 1.69 (95% CI, 1.15-2.49) for emphysema, 1.57 (95% CI, 1.15-2.13) for COPD, and 1.31 (95% CI, 1.01-1.71) for asthma.

**Conclusions and Relevance:**

This cohort study found that e-cigarette use was associated with an increased risk of developing respiratory disease independent of cigarette smoking. These findings add important evidence on the risk profile of novel tobacco products.

## Key Points

**Question:**

Is electronic cigarette (e-cigarette) use associated with incidence of self-reported respiratory disease compared with no use?

**Findings:**

In this nationally representative cohort of 21 618 US adults from the Population Assessment of Tobacco and Health study, former and current use of e-cigarettes were associated with increased risk of developing a respiratory condition compared with no prior use of e-cigarettes, independent of other tobacco product use.

**Meaning:**

These findings suggest that users of e-cigarettes may experience increased rates of respiratory conditions independent of other tobacco product use.

## Teaser

This cohort study examines the association of electronic cigarette use with incidence of respiratory conditions among participants of the Population Assessment of Tobacco and Health Study.

## Introduction

The use of electronic cigarettes (e-cigarettes) has increased dramatically since their introduction to the US market in 2007.^1,2,3^ While a 2018 National Academies of Sciences, Engineering, and Medicine report^4^ found substantial evidence that exposure to toxic substances from e-cigarettes is significantly lower compared with combustible cigarettes, recent studies and case reports suggest that e-cigarettes may present their own unique health risks, including to the respiratory system.^5,6,7,8^ e-Cigarettes are known to contain harmful or potentially harmful constituents, including volatile organic compounds, heavy metals, and ultrafine particles.^9,10,11,12^ e-Cigarette use has been associated with increased airway resistance, impairment of the immune system, and increased cytotoxic effects in the lungs.^13,14,15^

Small short-term studies conducted in vitro, in vivo, and in clinical settings have documented acute adverse pulmonary effects on organ and cellular health with e-cigarette use^13,16,17,18,19,20,21,22,23,24,25,26^; e-cigarette use has also been associated with the outbreak of e-cigarette or vaping product use–associated lung injury in the US.^8,27,28^ Despite existing evidence, the long-term effects of e-cigarette use on clinical respiratory end points remain unclear. Although a handful of cross-sectional studies have reported that e-cigarette use was associated with respiratory symptoms (eg, wheezing and related respiratory symptoms) and clinical outcomes. including asthma and chronic obstructive pulmonary disease (COPD),^29,30,31,32,33^ the causality of the association could not be established given the cross-sectional design. A 2019 longitudinal study by Bhatta and Glantz^34^ using Population Assessment of Tobacco and Health (PATH) study waves 1 through 3 found an association of e-cigarette use with a composite respiratory disease outcome. However, the study by Bhatta and Glantz^34^ did not consider the timing of respiratory events over follow-up and was underpowered to evaluate specific respiratory conditions. Additionally, the study included only crude adjustment for cigarette smoking history and did not examine the sensitivity of findings to potential reverse causal biases associated with preclinical disease.

In this study, we used data from PATH study waves 1 through 4 to examine the association of e-cigarette use with incident respiratory conditions, including COPD, emphysema, chronic bronchitis, and asthma. We incorporated information on the timing of respiratory events, included comprehensive adjustment for cigarette smoking history and use of other tobacco products, and conducted extensive sensitivity analyses to evaluate the robustness of the findings to potential reverse causality associated with preclinical disease. We hypothesized that adults who use e-cigarettes have higher risks of developing respiratory conditions compared with those who do not use e-cigarettes, and that the risks persist even after adjusting for smoking history and other important confounders.

## Methods

The PATH study design and protocol were approved by Westat's institutional review board, and participants provided written informed consent prior to participation.^35^ Use of the public-use PATH files for this cohort study was deemed exempt from review by the Boston University Medical Center institutional review board. This study followed the Strengthening the Reporting of Observational Studies in Epidemiology (STROBE) reporting guideline.

### Study Setting

The PATH study was initiated in 2013 by the National Institutes of Health and Food and Drug Administration as an annual longitudinal survey with a nationally representative sample of adults aged 18 years or older.^36,37^ The study oversamples tobacco users, young adults aged 18 to 24 years, and African American adults. Wave 1 data were collected from September 12, 2013, through December 14, 2014, and the most recent wave (ie, wave 4) of data collection was performed from December 1, 2016, to January 3, 2018. The PATH study offers the most comprehensive collection of tobacco- and e-cigarette–related questions among existing national surveys, to our knowledge. Details of the PATH study have been described elsewhere.^36^ For this study, we used data from the wave 1 cohort of adults, and the prospective follow-up at waves 2 to 4 from public use files. Youth who aged into the adult sample during follow-up and replenishment into the sample of adults at wave 4 were not included. Retention rates for the wave 1 cohort were 83.2% at wave 2, 78.4% at wave 3, and 73.5% at wave 4. We excluded individuals who reported a history of a respiratory condition (ie, COPD, emphysema, chronic bronchitis, or asthma) at wave 1 (baseline) in addition to those who were lost to follow-up by wave 2 and those with missing data on e-cigarette use behavior. For each specific respiratory condition, analytic sample sizes were dependent on the respective condition considered.

### Assessment of e-Cigarette Use

Individuals were asked a series of questions regarding their current tobacco use behaviors and tobacco use history at wave 1. Respondents were considered current e-cigarette users if they ever used an e-cigarette and currently used e-cigarettes every day or some days. Those who reported that they had ever used an e-cigarette but reported not using the product currently were defined as former users. Respondents who reported no prior e-cigarette use were considered never users. Ever e-cigarette users consisted of former and current e-cigarette users.

We further divided current e-cigarette use by some days vs every day, age of first use of e-cigarettes (<25 years, 25 to 44 years, ≥45 years). Former and current e-cigarette use was further divided into former experimental, former established, current experimental, and current established. Experimental use was defined by having never used e-cigarettes fairly regularly, and established use was defined as having used e-cigarettes fairly regularly.

### Respiratory Conditions

Adults were asked about their current health and medical history at each wave, including whether they had previously been diagnosed with a respiratory condition, including COPD, chronic bronchitis, emphysema, or asthma, from a physician or other health professional in the past 12 months. Among those with no history of respiratory disease at baseline, a confirmatory answer to specific respiratory conditions over follow-up defined an incident respiratory condition. In addition to assessing each respiratory condition separately, we generated a composite variable of having any lung or respiratory disease (reported ≥1 conditions). The survey instruments to assess respiratory disease have been validated against direct clinical observation and widely used in epidemiological studies.^38,39,40^

### Covariates

We incorporated covariates on age, sex, self-reported race/ethnicity (ie, non-Hispanic White, non-Hispanic Black, non-Hispanic other, or Hispanic); education (<high school, high school or GED, some college, or ≥bachelor’s degree); US census region (ie, Northeast, Midwest, South, or West); ever used heroin, inhalants, or hallucinogen; self-reported body mass index (BMI; calculated as weight in kilograms divided by height in meters squared); and self-reported health conditions (ie, hypertension, high cholesterol, heart failure, heart attack, stroke, or diabetes). We also incorporated data on cigarette smoking history (ie, never; former use, quit <5 years ago; former use, quit 5-20 years ago; former use, quit ≥20 years ;ago or more; current use, <5 pack-years; current use, 5-20 pack-years; or current use, ≥20 pack years); and ever use of other combustible tobacco products, including cigar, cigarillo, pipe tobacco, or hookah.

### Statistical Analysis

Reported numbers are unweighted and percentages are weighted using PATH sample weight. Poisson regression models were used to estimate incident rates and incident rate ratios (IRRs) along with 95% CIs. Respondents contributed follow-up time from baseline (wave 1) to the wave of diagnosis with a respiratory condition, loss to follow-up, or wave 4 (the end of follow-up), whichever came first. For the composite outcome, person-waves of exposure were calculated based on time to the first reported respiratory condition. IRRs indicate the relative risk of incident respiratory conditions for e-cigarette users compared with nonusers. We examined the association of e-cigarette use with incident respiratory conditions with sequential adjustment for covariates. In the first model, we adjusted for sociodemographic characteristics, including age, sex, and race/ethnicity. In the second model, we additionally adjusted for educational attainment, US census region, cigarette smoking history, ever use of other combustible products (ie, pipe, cigar, cigarillo, or hookah), ever use of illicit substances (ie, heroin, inhalants, or hallucinogens), BMI, and self-reported health conditions (eg, hypertension, cholesterol, heart failure, stroke, diabetes).

To address potential reverse causality associated with preclinical disease, we repeated the analyses limiting the sample to healthy respondents. We developed 2 subsamples of healthy respondents, first restricting to respondents with excellent, great, or good self-rated health and second restricting to respondents free of all cardiopulmonary chronic conditions at baseline.

We used multiple imputation by chained equations (5 imputations) to account for missing data in exposure and covariates. We accounted for complex survey design using the wave 2 sample weights to compensate for different probabilities of selection, nonresponse, possible deficiencies in the sampling frame, and attrition.^36^ We accounted complex survey design using wave 2 sample weights to compensate for different probabilities of selection, nonresponse, possible deficiencies in the sampling frame, and attrition. We used wave 2 sample weights rather than the wave 4 all-wave panel weights in this study given our inclusion of respondents who contributed at least 1 person-wave of follow-up. All analyses were conducted in Stata statistical software version 16 (StataCorp). *P* values were 2-sided, and statistical significance was set at *P* < .05. Analyses were conducted from February to July 2020.

We conducted secondary analyses assessing the influence of e-cigarette use intensity and effect modification by sex. Moreover, a series of sensitivity analyses to check the robustness of our results on the composite outcome were performed. First, we further adjusted for secondhand smoke exposure at home or work, marijuana use, childhood secondhand smoke exposure, and ever use of any noncombustible tobacco, including snus, dissolvable, and smokeless tobacco. Second, we re-estimated all models restricting analyses to individuals without missing data (ie, listwise deletion) rather than multiple imputation. Third, we repeated the analyses using PATH study replicated weights, calculated using Fay variant of balanced repeated replication.^36,41^ Fourth, we repeated the analyses applying wave 4 all-wave panel weight. Fifth, we restricted our analyses to respondents who never smoked cigarette or smoked fewer than 100 cigarettes in their lifetime. Finally, we further restricted our analyses to respondents who never used cigarette and other tobacco products, including hookah, cigar, cigarillo, smokeless, dissolvable, and snus.

## Results

### Baseline Characteristics and e-Cigarette Use Behavior

Among 21 618 respondents included in the analyses (eFigure 1 in the Supplement), 11 017 (49.1%) were men, 12 969 (65.2%) were non-Hispanic White, 3216 (11.5%) were non-Hispanic Black, 3839 (15.5%) were Hispanic, and 1594 (7.9%) were non-Hispanic other. Mean (SD) pack-years at baseline was 6.7 (25.8) pack-years. After survey weighting, respondents were evenly distributed by among age groups (18-24 years: 6088 respondents [13.1%]; 25-34 years: 4334 respondents [18.3%]; 35-44 years: 3436 respondents [17.2%]; 45-54 years: 3282 respondents [18.2%]; 66-64 years: 2569 respondents [16.4%]; ≥65 years: 1907 respondents [16.9%]). At baseline, 7405 respondents (16.8%) were ever e-cigarette users, among them 2329 respondents (5.2%) were current users (Table 1). Compared with never users, current e-cigarette users were younger (age 18-24 years: 3540 respondents [10.8%] vs 730 respondents [21.9%]) and were more likely to use other combustible tobacco products (never cigarette-smokers: 9138 respondents [72.1%] vs 401 respondents [16.0%]) and illicit drugs (750 respondents [4.0%] vs 353 respondents [15.8%]) (Table 1).

**Table 1.** Sample Characteristics by e-Cigarette Use Status at Baseline

Characteristics	Respondents, No. (%)^a^			
	Total (N = 21 618)	e-Cigarette use status^b^		
		Never (n = 14 213)	Former (n = 5076)	Current (n = 2329)
Cigarette smoking status				
Never	10 930 (64.1)	9138 (72.1)	1391 (27.0)	401 (16.0)
Former, y				
Quit <5	1271 (4.3)	448 (2.8)	550 (11.4)	273 (13.1)
Quit 5-20	1031 (6.7)	804 (7.2)	196 (4.9)	31 (1.6)
Quit ≥20	1006 (8.9)	941 (10.4)	49 (1.4)	12 (0.6)
Current, pack-years				
<5	2246 (4.7)	669 (1.8)	1013 (18.1)	564 (22.0)
5-20	2592 (6.3)	985 (2.9)	1007 (21.2)	600 (28.2)
≥20	1925 (5.1)	866 (2.8)	688 (16.1)	371 (18.5)
Pack-years, mean (95% CI)	6.7 (6.3-7.0)	5.6 (5.2-6.0)	11.6 (10.8-12.3)	13.4 (12.2-14.6)
Men	11 017 (49.1)	7080 (47.7)	2701 (56.8)	1236 (55.8)
Age, y				
18-24	6088 (13.1)	3540 (10.8)	1818 (25.4)	730 (21.9)
25-34	4334 (18.3)	2421 (15.9)	1324 (30.4)	589 (29.9)
35-44	3436 (17.2)	2238 (17.0)	797 (17.9)	401 (18.8)
45-54	3282 (18.2)	2343 (19.1)	608 (13.4)	331 (15.2)
55-64	2569 (16.4)	1970 (17.7)	383 (9.1)	216 (10.8)
≥65	1907 (16.9)	1700 (19.6)	146 (3.8)	61 (3.4)
Race/ethnicity				
Non-Hispanic White	12 969 (65.2)	8197 (64.7)	3212 (66.7)	1560 (70.4)
Non-Hispanic Black	3216 (11.5)	2391 (11.6)	608 (11.4)	217 (8.9)
Hispanic	3839 (15.5)	2613 (15.7)	868 (15.1)	358 (12.9)
Non-Hispanic other	1594 (7.9)	1012 (8.0)	388 (6.9)	194 (7.8)
Education				
<High school	2706 (11.0)	1794 (10.9)	623 (11.0)	289 (11.4)
High school or GED	6419 (29.0)	3893 (27.8)	1718 (34.8)	808 (35.5)
Some college	7560 (30.9)	4641 (29.3)	1981 (37.7)	938 (39.5)
Bachelor or above	4855 (29.2)	3827 (31.9)	740 (16.4)	288 (13.6)
Census region				
Northeast	3187 (18.1)	2198 (18.6)	736 (17.2)	253 (13.0)
Midwest	5257 (21.3)	3330 (20.9)	1322 (23.6)	605 (22.8)
South	8257 (37.1)	5426 (36.9)	1918 (37.3)	913 (39.6)
West	4917 (23.5)	3259 (23.6)	1100 (22.0)	558 (24.7)
Ever smoked				
Cigar	8376 (31.5)	4396 (26.4)	2714 (56.4)	1266 (56.3)
Cigarillo	9199 (28.8)	4397 (21.4)	3326 (65.9)	1476 (64.0)
Pipe	4738 (17.7)	2369 (14.6)	1562 (31.8)	807 (35.0)
Hookah	8071 (16.4)	3527 (10.5)	2446 (45.9)	1098 (44.6)
Ever drug use^c^	1861 (5.9)	750 (4.0)	758 (15.4)	353 (15.8)
BMI, mean (95% CI)	27.8 (27.7-27.9)	27.9 (27.8-28.0)	27.5 (27.3-27.6)	27.5 (27.2-27.8)
Self-reported conditions				
Hypertension	4414 (25.8)	3159 (27.3)	852 (18.9)	403 (18.5)
High cholesterol	3490 (22.1)	2602 (23.8)	611 (13.8)	277 (13.2)
Heart failure	177 (1.0)	138 (1.1)	23 (0.5)	16 (0.7)
Stroke	286 (1.6)	201 (1.6)	66 (1.5)	19 (0.9)
Heart attack	315 (1.9)	248 (2.1)	47 (1.1)	20 (1.2)
Diabetes	2205 (13.0)	1604 (13.9)	412 (8.6)	189 (8.3)

Abbreviations: BMI, body mass index (calculated as weight in kilograms divided by height in meters squared); e-cigarette, electronic cigarette; GED, general education development.

^a^ Numbers are unweighted, and percentages are weighted. The number of respondents with missing values are 617 respondents for pack-year, 2 respondents for age, 78 respondents for education, 780 respondents for ever cigar use, 609 respondents for ever cigarillo use, 28 respondents for ever pipe use, 21 respondents for ever hookah use, 36 respondents for each health condition history, 22 respondents for ever had diabetes, 61 respondents for ever used heroin or other drugs, and 340 respondents for BMI.

^b^ Current use was defined as now uses e-cigarette every day or some days and former use was defined as ever used e-cigarettes, even 1 or 2 puffs, and currently not using e-cigarettes.

^c^ Includes ever use any of heroin, inhalants, or hallucinogen.

### e-Cigarette Use and Incident Respiratory Conditions

During follow-up, there were 804 newly reported cases of asthma, 336 newly reported cases of emphysema, 573 newly reported cases of COPD, and 948 newly reported cases of chronic bronchitis. There were 1460 respondents (6.8%) with at least 1 reported respiratory disease. Table 2 presents the association of e-cigarette use with incident respiratory conditions. In the multivariable-adjusted models, ever e-cigarette use was associated with increased risk of developing any respiratory condition (IRR, 1.28; 95% CI, 1.10-1.48), chronic bronchitis (IRR, 1.33; 95% CI, 1.12-1.59), emphysema (IRR, 1.54; 95% CI, 1.12-2.12), COPD (IRR, 1.62; 95% CI, 1.28-2.04), and asthma (IRR, 1.24; 95% CI, 1.01-1.53). Among ever e-cigarette users, the risks were similar for current vs former e-cigarette users. For the composite outcome, former e-cigarette users had a 28% (95% CI, 9%-51%) increased risk of developing respiratory disease, while current e-cigarette users had 31% (95% CI, 8%-59%) increased risk compared with respondents with no prior e-cigarette use.

**Table 2.** Associations of e-Cigarette Use With Respiratory Conditions

Condition	e-Cigarette status^a^		Among ever users of e-cigarettes^b^	
	Never	Ever	Former	Current
Any respiratory condition (n = 21 640)^c^				
Incident cases, No.	838	622	412	210
Multivariable-adjusted incidence rate, per 1000 person-years (95% CI)^d^	20.41 (18.65-22.17)	26.06 (22.63-29.49)	26.12 (22.34-29.89)	26.68 (21.83-31.52)
Age-, sex-, and race/ethnicity-adjusted IRR (95% CI)	1 [Reference]	2.14 (1.87-2.44)	2.09 (1.81-2.42)	2.23 (1.87-2.67)
Multivariable-adjusted IRR (95% CI)^d^	1 [Reference]	1.28 (1.10-1.48)	1.28 (1.09-1.51)	1.31 (1.08-1.59)
Chronic bronchitis (n = 25 202)				
No. of incident cases, No.	529	419	274	144
Multivariable-adjusted incidence rate, per 1000 person-years (95% CI)^d^	12.18 (10.86-13.50)	16.23 (13.68-18.79)	15.96 (13.17-18.75)	16.26 (12.72-19.80)
Age-, sex-, and race/ethnicity-adjusted IRR (95% CI)	1 [Reference]	2.20 (1.88-2.59)	2.16 (1.80-2.58)	2.27 (1.83-2.81)
Multivariable-adjusted IRR (95% CI)^d^	1 [Reference]	1.33 (1.12-1.59)	1.31 (1.08-1.58)	1.33 (1.06-1.67)
Emphysema (n = 23 686)				
No. of incident cases, No.	181	155	92	62
Multivariable-adjusted incidence rate, per 1000 person-years (95% CI)^d^	3.74 (3.02-4.46)	5.76 (4.08-7.43)	5.35 (3.64-7.05)	6.35 (4.00-8.69)
Age-, sex-, and race/ethnicity-adjusted IRR (95% CI)	1 [Reference]	4.02 (3.06-5.27)	3.73 (2.74-5.09)	4.57 (3.26-6.41)
Multivariable-adjusted IRR (95% CI)^d^	1 [Reference]	1.54 (1.12-2.12)	1.42 (1.01-2.01)	1.69 (1.15-2.49)
COPD (n = 23 236)				
No. of incident cases, No.	298	275	181	94
Multivariable-adjusted incidence rate, per 1000 person-years (95% CI)^d^	6.48 (5.55-7.42)	10.49 (8.32-12.65)	10.72 (8.33-13.11)	10.13 (7.24-13.03)
Age-, sex-, and race/ethnicity-adjusted IRR (95% CI)	1 [Reference]	5.03 (4.12-6.14)	5.05 (4.05-6.28)	5.09 (3.87-6.68)
Multivariable-adjusted IRR (95% CI)^d^	1 [Reference]	1.62 (1.28-2.04)	1.66 (1.29-2.12)	1.57 (1.15-2.13)
Asthma (n = 20 914)				
No. of incident cases, No.	460	344	224	119
Multivariable-adjusted incidence rate, per 1000 person-years (95% CI)^d^	10.73 (9.46-12.00)	13.31 (10.90-15.71)	12.82 (10.27-15.37)	14.14 (10.69-17.59)
Age-, sex-, and race/ethnicity-adjusted IRR (95% CI)	1 [Reference]	1.76 (1.48-2.09)	1.68 (1.39-2.04)	1.93 (1.52-2.45)
Multivariable-adjusted IRR (95% CI)^d^	1 [Reference]	1.24 (1.01-1.53)	1.19 (0.95-1.50)	1.32 (1.01-1.72)

Abbreviations: COPD, chronic obstructive pulmonary disease; e-cigarette, electronic cigarette; IRR, incidence rate ratio.

^a^ Baseline e-cigarette use status was dichotomized as those who never used e-cigarette, even 1 or 2 puffs (never users) vs who ever used (ever users).

^b^ Current use was defined as now uses e-cigarette every day or some days, and former use was defined as ever used e-cigarettes, even 1 or 2 puffs, and currently not using e-cigarettes. The reference category was never use.

^c^ Includes COPD, chronic bronchitis, emphysema, and asthma.

^d^ In addition to adjusting age, sex, and race/ethnicity, adjusted for education; US census region, cigarette smoking status, ever smoked cigar, ever smoked cigarillo, ever smoked pipe, ever smoked hookah, ever used heroin or inhalants or hallucinogen, ever diagnosed with health conditions (ie, hypertension, cholesterol, heart failure, stroke, and diabetes), and body mass index.

In the analyses restricted to respondents with good, great, or excellent health, individuals with former e-cigarette use had a 21% (95% CI, 0%-47%) increased risk of developing a respiratory condition compared with individuals with no prior e-cigarette use, controlling for all the covariates, and those with current e-cigarette use had a 43% (95% CI, 14%-80%) increased risk (Table 3). Among respondents with no chronic conditions at baseline, current e-cigarette use was associated with a 40% (95% CI, 9%-79%) increased risk of developing a respiratory condition (Figure).

**Table 3.** Associations of e-Cigarette Use With Respiratory Conditions Among Healthy Respondents^a^

Subgroup	e-Cigarette status^b^		Among ever users of e-cigarettes^c^	
	Never	Ever	Former	Current
Excluding respondents with any chronic conditions (n = 14 865)^d^				
Incident cases, No.	445	363	229	134
Multivariable-adjusted incidence rate, per 1000 person-years (95% CI)^e^	16.59 (14.62-18.56)	19.85 (16.57-23.14)	18.36 (14.94-21.77)	23.18 (17.92-28.42)
Age-, sex-, race/ethnicity-adjusted IRR (95% CI)	1 [Reference]	1.91 (1.60-2.27)	1.73 (1.43-2.10)	2.30 (1.83-2.90)
Multivariable-adjusted IRR (95% CI)^e^	1 [Reference]	1.20 (0.99-1.45)	1.11 (0.90-1.37)	1.40 (1.09-1.79)
Excluding respondents with fair or poor health (n = 18 649)				
No. of incident cases, No.	612	433	276	157
Multivariable-adjusted incidence rate, 1000 person-years^e^	17.29 (15.55-19.03)	21.95 (18.53-25.37)	20.94 (17.31-24.57)	24.73 (19.42-30.03)
Age-, sex-, race/ethnicity-adjusted IRR (95% CI)	1 [Reference]	2.07 (1.78-2.42)	1.92 (1.61-2.28)	2.40 (1.94-2.95)
Multivariable-adjusted IRR (95% CI)^e^	1 [Reference]	1.27 (1.07-1.51)	1.21 (1.00-1.47)	1.43 (1.14-1.80)

Abbreviations: e-cigarette, electronic cigarette; IRR, incidence rate ratio.

^a^ Respiratory disease includes chronic obstructive pulmonary disease, chronic bronchitis, emphysema, and asthma.

^b^ Baseline e-cigarette use status was dichotomized as those who never used e-cigarette, even 1 or 2 puffs (never users) vs who ever used (ever users).

^c^ Current use was defined as now uses e-cigarette every day or some days, and former use was defined as ever used e-cigarette, even 1 or 2 puffs, and currently not using e-cigarette. The reference category was never use.

^d^ Analyses restricted to respondents who had no self-reported chronic conditions, including any general health conditions (ie, hypertension, high cholesterol, heart attack, heart failure, stroke, and diabetes) or respiratory conditions (chronic obstructive pulmonary disease, emphysema, chronic bronchitis, and asthma).

^e^ In addition to adjusting age, sex, and race/ethnicity, adjusted for education; US census region, cigarette smoking status, ever smoked cigar, ever smoked cigarillo, ever smoked pipe, ever smoked hookah, ever used heroin or inhalants or hallucinogen, ever diagnosed with health conditions (ie, hypertension, cholesterol, heart failure, stroke, and diabetes), and body mass index.

**Figure.**
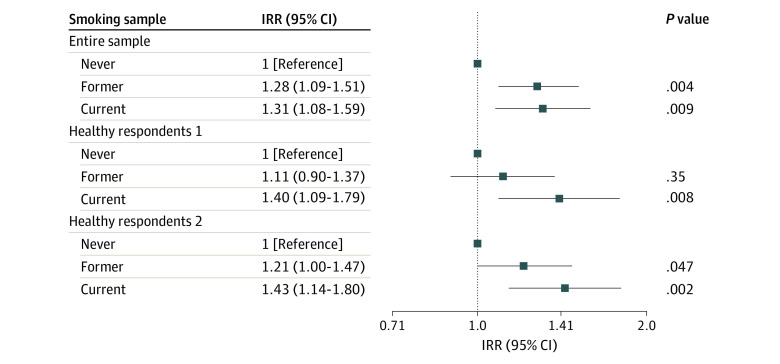
Associations of e-Cigarette Use With the Risk of Respiratory Disease^a^ Healthy respondents 1 indicates individuals who had no self-reported chronic conditions, including any general health (ie, hypertension, high cholesterol, heart attack, heart failure, stroke, and diabetes) or respiratory-specific conditions (ie, chronic obstructive pulmonary disease, emphysema, chronic bronchitis, and asthma); healthy respondents 2, individuals whose self-rated overall physical health were good, great, or excellent; and IRR, incidence rate ratio. ^a^Includes chronic obstructive pulmonary disease, chronic bronchitis, emphysema, and asthma.

In the exploratory analyses further dividing e-cigarette users by intensity of current use, starting age, and experimental vs established use, the highest incident rate for respiratory disease was observed in groups with current every-day use, initiated e-cigarettes before age 25 years, and established use (eFigure 2 in the Supplement). We found no significant interactions with sex. Sensitivity analyses, further adjustment for noncombustible tobacco use, secondhand smoke exposure, marijuana use, and childhood secondhand smoking exposure, applying listwise deletion of missing cases, using replicate weights, using panel weights, excluding cigarette smokers, and excluding cigarette or other tobacco product users yielded similar findings to the main results (eTable in the Supplement).

## Discussion

This cohort study adds to the evolving literature on the associations of e-cigarette use with respiratory health. Using data from a large nationally representative cohort of adults, we found that e-cigarette use was associated with increased risk of asthma, COPD, emphysema, and chronic bronchitis, independent of cigarette smoking and other combustible tobacco product use.

Our findings on clinical outcome were consistent with studies assessing in vivo biomarkers of e-cigarette exposure in animal subjects, human participants, and population studies.^9,30,34,42^ Studies have documented that exclusive e-cigarette use may be associated with higher exposure to harmful and potentially harmful constituents compared with tobacco nonuse.^43,44^ The potential mechanisms of the association of e-cigarette exposure with pulmonary diseases include pulmonary inflammation, increased oxidative stress, and inhibited immune response.^45^ Animal studies have generated substantial evidence on e-cigarette exposure and emphysematous lung destruction, loss of pulmonary capillaries, reduced small airway function, and airway hyperresponsiveness,^46,47,48,49^ suggesting the plausibility of e-cigarettes causing chronic lung diseases.

While the associations with e-cigarette use were largely consistent across respiratory conditions, outcomes associated with e-cigarette use may vary according to specific respiratory conditions. Asthma is an allergic disease that often develops in childhood, usually characterized by chronic airway inflammation and reversible airflow obstruction.^50^ In contrast, COPD typically develops in mid- to later-life, characterized physiologically by incompletely reversible airflow limitation from a progressively decline in lung function.^51^ The potentially distinctive role of e-cigarette on these specific respiratory conditions needs to be clarified in future investigations.

Reverse causality in the association of e-cigarette use with respiratory outcomes is a potentially serious bias in studies of the epidemiology of tobacco use, which may limit the validity of study findings. Chronic respiratory diseases can take substantial time to develop before resulting in clinical manifestations and diagnosis by a health care practitioner. However, prior to diagnosis, preclinical respiratory conditions may influence tobacco use behaviors, including motivating cigarette smokers to switch to e-cigarettes or quit tobacco use altogether. This reverse causal bias has the potential to attenuate the association of e-cigarette use with development of respiratory outcomes, creating the appearance that e-cigarette use is associated with no or little excess risk of disease. In this study, we sought to address this potential bias through adjustment for a comprehensive set of health conditions and by repeating analyses after restricting the sample to healthy respondents and those free of chronic conditions. In the latter analyses, we observed a significant association of e-cigarette use with respiratory disease, consistent with the findings of our primary analysis. There was no statistically significant difference in risks between current and former e-cigarette users in a post hoc test.

Few population studies have examined the association of e-cigarette use with respiratory disease. A cross-sectional study among 705 159 respondents in Behavioral Risk Factor Surveillance System 2016 and 2017 reported that current e-cigarette use was associated with 75% higher odds of reporting COPD among never cigarette smokers.^33^ Using the same data from the PATH study, Bhatta and Glantz^34^ reported a 31% increase in the risk of reporting respiratory disease among former e-cigarette users and 29% increase among current e-cigarette users adjusted for demographic characteristics, combustible tobacco smoking, and clinical variables. Our longitudinal results are consistent with the findings of prior population studies.^33,34^ With a more refined study design assessing multiple respiratory conditions and extensive sensitivity checks to mitigate bias from reverse causation and residual confounding by cigarette smoking and other tobacco product use, our results strengthen the evidence of the potential role of e-cigarette use in pulmonary disease pathogenesis. The findings may be used to inform counseling of patients on the potential risks of e-cigarette use.

Globally, COPD is a significant public health problem and leading cause of morbidity and mortality.^52,53^ In the US, COPD affects more that 14.8 million adults and is the fourth leading cause of death.^54^ In addition, more than 25 million people in the US have asthma.^55,56^ Combined, asthma and COPD cause more than 1 million hospitalizations and more than 15 million lost work days.^57^ Our findings highlight the potential respiratory harms associated with e-cigarette use and the importance of developing regulations to combat their increasing use.

This study has several strengths, including use of a large and nationally representative cohort. Our study also included careful adjustment for cigarette smoking and other tobacco product use, extensive sensitivity checks for reverse causation, and detailed analysis of e-cigarette use patterns and intensity for multiple respiratory conditions and among important demographic subgroups.

### Limitations

This study has some limitations. First, we relied on self-reported measures of e-cigarette and other tobacco product use, which may be subject to recall bias. Although these survey instruments have been validated and widely used in epidemiologic studies, some misclassification may be inevitable, which may lead to weakened associations. Second, we relied on self-reported data on the diagnosis of respiratory diseases. Our results indicated that e-cigarette users experienced symptoms of airway and alveolar disease; however, changes in pulmonary function tests indicative of COPD may not become apparent until years after e-cigarette exposure. Future studies should track serial pulmonary function tests to understand the full risk of e-cigarette–attributable lung disease, such as COPD. Third, the data were observational in nature, and the follow-up period was relatively short; thus, the study could not establish causality. Fourth, we did not consider time-varying exposure or confounding. Incorporating time-varying exposure may complicate the interpretation of the findings if preclinical respiratory symptoms that emerge over follow-up in turn lead to changes in tobacco use behaviors. Future studies should consider the use of more flexible longitudinal modeling strategies, such as marginal structural models.^58^ Fifth, a few important e-cigarette device characteristics were either not available or incomplete (eg, device generation and flavor) in the initial wave, precluding an analysis of association modification by these factors. Given the importance of these characteristics in determining levels of nicotine and toxicants exposure, future investigations to examine the influence of device characteristics are warranted.

## Conclusions

In this large nationally representative cohort study of US adults, e-cigarette use was associated with significantly increased risk of major respiratory diseases, independent of cigarette smoking and other tobacco product use. These findings add important evidence on the risk profile of novel tobacco products with respect to respiratory outcomes.

## Notes

Supplement. **eTable.** Sensitivity Analysis of Associations of e-Cigarette Use With Respiratory Disease **eFigure 1.** Selection of Study Participants From the PATH Study From 2013 to 2018 to Examine Respiratory Disease **eFigure 2.** Associations of e-Cigarette Use With the Risk of Respiratory Disease
