# Supplementary material for: Association of Electronic Cigarette Use With Incident Respiratory Conditions Among US Adults From 2013 to 2018
Source: JAMA Netw Open. 2020 Nov 12;3(11):e2020816. doi: 10.1001/jamanetworkopen.2020.20816 (PMC7662143; doi:10.1001/jamanetworkopen.2020.20816)
Supplement: Supplement. — eTable. Sensitivity Analysis of Associations of e-Cigarette Use With Respiratory Disease eFigure 1. Selection of Study Participants From the PATH Study From 2013 to 2018 to Examine Respiratory Disease eFigure 2. Associations of e-Cigarette Use With the Risk of Respiratory Disease [file jamanetwopen-e2020816-s001.pdf]

# Supplemental Online Content

Xie W, Kathuria H, Galiatsatos P, et al. Association of electronic cigarette use with incident respiratory conditions among US adults from 2013 to 2018. *JAMA Netw Open*. 2020;3(11):e2020816. doi:10.1001/jamanetworkopen.2020.20816

**eTable.** Sensitivity Analysis of Associations of e-Cigarette Use With Respiratory Disease

**eFigure 1.** Selection of Study Participants From the PATH Study From 2013 to 2018 to Examine Respiratory Disease

**eFigure 2.** Associations of e-Cigarette Use With the Risk of Respiratory Disease

This supplemental material has been provided by the authors to give readers additional information about their work.

© 2020 Xie W et al. *JAMA Network Open.*

**eTable.** Sensitivity Analysis of Associations of e-Cigarette Use With Respiratory Disease

| | E-cigarette Status [b] | | | Among Ever Users [c] | |
|---|---|---|---|---|---|
| | Never | Ever | | Former | Current |
| *Entire sample (n=21 640)* | | | | | |
| No. of incident cases | 838 | 622 | | 412 | 210 |
| Multivariable-adjusted IRR (95% CI)[d] | 1 [Reference] | 1.28 (1.10-1.48) | | 1.28 (1.09-1.51) | 1.31 (1.08-1.59) |
| *Sensitivity check 1 - Further control [e] (n=21 640)* | | | | | |
| No. of incident cases | 838 | 622 | | 412 | 210 |
| Multivariable-adjusted IRR (95% CI) [e] | 1 [Reference] | 1.26 (1.08-1.46) | | 1.25 (1.06-1.47) | 1.27 (1.05-1.55) |
| *Sensitivity check 2 - List-wise deletion [f] (n=19 862)* | | | | | |
| No. of incident cases | 743 | 571 | | 384 | 187 |
| Multivariable-adjusted IRR (95% CI)[d] | 1 [Reference] | 1.30 (1.11-1.52) | | 1.31 (1.10-1.54) | 1.28 (1.04-1.58) |
| *Sensitivity check 3 - List-wise deletion with replicate weights [g] (n=19 862)* | | | | | |
| No. of incident cases | 743 | 571 | | 384 | 187 |
| Multivariable-adjusted IRR (95% CI)[d] | 1 [Reference] | 1.30 (1.10-1.54) | | 1.31 (1.09-1.57) | 1.28 (1.04-1.59) |
| *Sensitivity check 4 - Wave 4 all-wave weights [h] (n=17 391)* | | | | | |
| No. of incident cases | 729 | 513 | | 350 | 181 |
| Multivariable-adjusted IRR (95% CI)[d] | 1 [Reference] | 1.21 (1.03-1.42) | | 1.18 (0.99-1.41) | 1.25 (1.01-1.55) |
| *Sensitivity check 5 - Excluding Cigarette Smokers [i] (n=10 946)* | | | | | |
| No. of incident cases | 438 | 118 | | 87 | 31 |
| Multivariable-adjusted IRR (95% CI)[d] | 1 [Reference] | 1.37 (1.05-1.79) | | 1.38 (1.03-1.84) | 1.35 (0.87-2.09) |
| *Sensitivity check 6 - Excluding Tobacco Users [j] (n=4892)* | | | | | |
| No. of incident cases | 275 | 21 | | 12 | 9 |
| Multivariable-adjusted IRR (95% CI)[d] | 1 [Reference] | 1.49 (0.88-2.51) | | 1.29 (0.65-2.56) | 2.10 (1.00-4.39) |

Abbreviations: CI, confidence interval; IRR, incidence rate ratio

[a] Respiratory disease includes COPD, emphysema, chronic bronchitis, and asthma

[b] Baseline e-cigarette use status was dichotomized as those who never used e-cigarette even one or two puffs (Never) vs who ever used (Ever).

[c] Current use - now uses e-cigarette every day, some days, Former - ever used e-cigarette even one or two puffs and currently not using e-cigarette. The reference category was also "never use".

[d] Adjusted for age (categories), sex (male/female), race/ethnicity (non-Hispanic white, non-Hispanic black, Hispanic, non-Hispanic other), education (less than high school, high school and GED, some college, bachelor or above); US census region (northeast, midwest, south, west), cigarette smoking status (never, former with quitted <5, 5-20 or ≥20 years, former-quitted >20 years, current with <5, 5-20, or ≥20 pack-years), ever smoked pipe (yes or no), ever smoked hookah (yes or no), ever used heroin or inhalants or hallucinogen (yes or no), ever diagnosed with cardiovascular conditions, including hypertension (yes or no) , cholesterol (yes or no), heart failure (yes or no), stroke (yes or no), diabetes (yes or no), body mass index ($kg/m^2$), ever smoked cigar (yes or no), ever smoked cigarillo (yes or no), and childhood secondhand smoke exposure (yes or no).

[e] Further controlled for noncombustible smoking (yes or no), and childhood secondhand smoking exposure (yes or no), current secondhand smoke exposure (no exposure, exposure either at work or at home, exposed both at work and at home), and marijuana use (never, former, current).

[f] List-wise deletion of cases with missing values; sample weight applied.

[g] List-wise deletion of cases with missing values; the balanced repeated replication (BRR) method with Fay adjustment (Fay = 0.3) was applied.

© 2020 Xie W et al. *JAMA Network Open.*

[h] Wave 4 all-wave panel weight was applied, missing data on any covariates were imputed with multiple imputation by chained equation.
[i] Analyses restricted to never smokers (i.e. never smoked cigarette or smoked less than 100 cigarettes in their life time).
[j] Analyses restricted to respondents who never smoked cigarette and never use other tobacco products including cigar, cigarillo, hookah, smokeless, snus, and dissolvable.

© 2020 Xie W et al. *JAMA Network Open.*

**eFigure 1.** Selection of Study Participants From the PATH Study From 2013 to 2018 to Examine Respiratory Disease

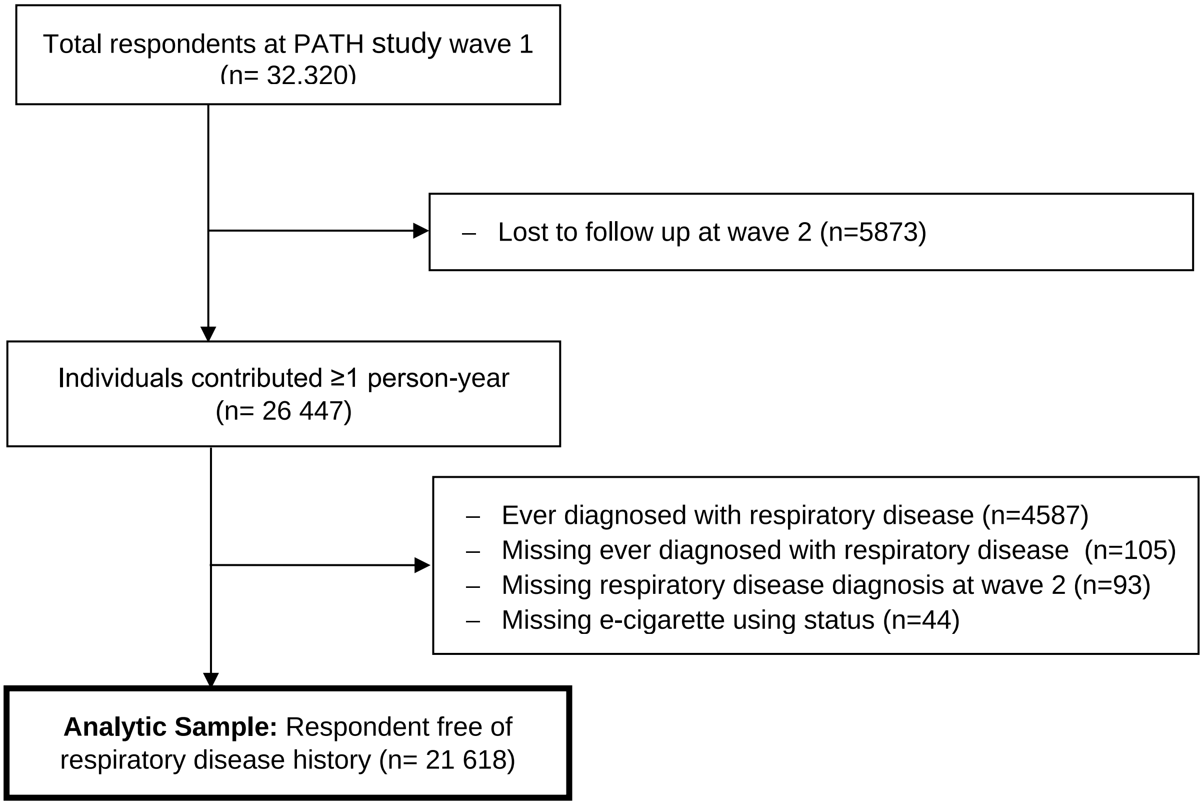


© 2020 Xie W et al. *JAMA Network Open.*

**eFigure 2.** Associations of e-Cigarette Use With the Risk of Respiratory Disease

## A. Current use intensity

| No. of cases | Incidence, per 1000 person-year | Adjusted IRR [b] |
|---|---|---|
| 838 | 20.40 (18.65-22.16) | 1 [Reference] |
| 412 | 25.89 (22.16-29.63) | 1.27 (1.08-1.49) |
| 167 | 26.04 (20.85-31.22) | 1.28 (1.03-1.57) |
| 43 | 28.10 (18.26-37.93) | 1.38 (0.96-1.97) |

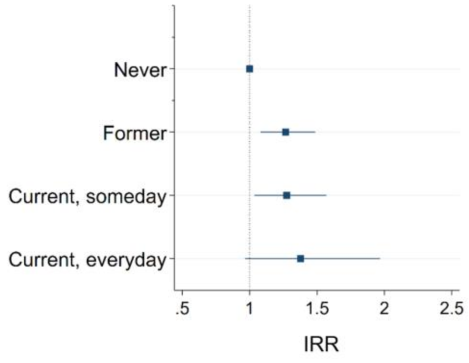


## B. Starting age

| No. of cases | Incidence, per 1000 person-year | Adjusted IRR [b] |
|---|---|---|
| 838 | 20.36 (18.61-22.11) | 1 [reference] |
| 412 | 26.05 (22.27-29.82) | 1.28 (1.09-1.50) |
| 67 | 32.20 (21.74-42.66) | 1.58 (1.14-2.20) |
| 70 | 24.72 (17.43-32.01) | 1.21 (0.90-1.65) |
| 72 | 25.80 (18.54-33.06) | 1.27 (0.95-1.70) |

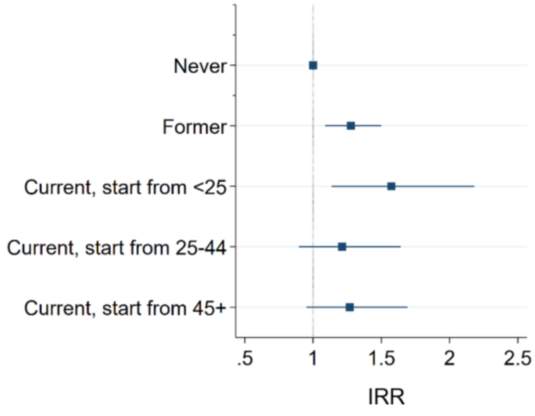


## C. Experimenting vs established

© 2020 Xie W et al. *JAMA Network Open.*

| No. of cases | Incidence, per 1000 person-year | Adjusted IRR [b] |
|---|---|---|
| 838 | 20.41 (18.65-22.16) | 1 [reference] |
| 371 | 25.75 (21.92-29.57) | 1.26 (1.07-1.49) |
| 40 | 27.27 (17.66-36.87) | 1.34 (0.93-1.91) |
| 118 | 25.02 (19.34-30.70) | 1.23 (0.97-1.55) |
| 92 | 28.62 (21.42-35.82) | 1.40 (1.08-1.82) |

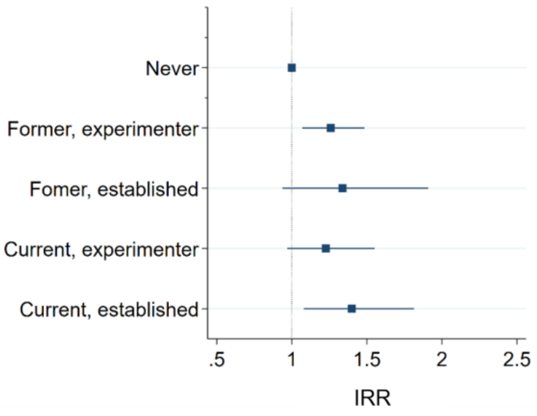


[a] Includes COPD, chronic bronchitis, emphysema and asthma

[b] IRR, incidence rate ratio; adjusted for age (categories), sex (male/female), race/ethnicity (non-Hispanic white, non-Hispanic black, Hispanic, non-Hispanic other), education (less than high school, high school and GED, some college, bachelor or above); US census region (northeast, midwest, south, west), cigarette smoking status (never, former with quitted <5, 5-20 or ≥20 years, former-quitted >20 years, current with <5, 5-20, or ≥20 pack-years), ever smoked cigar (yes or no), ever smoked cigarillo (yes or no), ever smoked pipe (yes or no), ever smoked hookah (yes or no), ever used heroin or inhalants or hallucinogen (yes or no), ever diagnosed with cardiovascular conditions, including hypertension (yes or no) , cholesterol (yes or no), heart failure (yes or no), stroke (yes or no), diabetes (yes or no), and body mass index (kg/m$^2$, continuous)

© 2020 Xie W et al. *JAMA Network Open.*
